# Ultra-processing markers are more prevalent in plant-based meat products as compared to their meat-based counterparts in a German food market analysis

**DOI:** 10.1017/S1368980023002458

**Published:** 2023-12

**Authors:** Kemja-Maria Metz, Nathalie Judith Neumann, Mathias Fasshauer

**Affiliations:** 1 Institute of Nutritional Science, Justus-Liebig University of Giessen, Goethestr. 55, Giessen, Hessen 35390, Germany; 2 Center for Sustainable Food Systems, Justus-Liebig University of Giessen, Giessen, Hessen, Germany

**Keywords:** Metabolic syndrome, NOVA classification, Nutrient composition, Plant-based meat products, Ultra-processed food

## Abstract

**Objective:**

To compare ultra-processing markers and nutrient composition in plant-based meat products (PBMP) with equivalent meat-based products (MBP).

**Design:**

A total of 282 PBMP and 149 MBP within 18 product categories were assessed. Based on the NOVA classification, 33 ultra-processing markers were identified and six ultra-processing bullet categories were defined, that is flavour, flavour enhancer, sweetener, colour, other cosmetic additives and non-culinary ingredients. The ingredient lists were analysed concerning these ultra-processing markers and ultra-processing bullet categories, as well as nutrient composition, for all PBMP and MBP. Differences between PBMP and MBP were assessed using chi-square and Mann-Whitney *U* tests, respectively.

**Setting:**

Cross-sectional analysis.

**Participants:**

282 PBMP and 149 MBP.

**Results:**

The percentage of ultra-processed food (UPF) items was significantly higher in PBMP (88 %) as compared to MBP (52 %) (*P* < 0·0001). The proportion of UPF items was numerically higher in 15 out of 18 product categories with differences in six categories reaching statistical significance (*P* < 0·05). Flavour, flavour enhancer, colour, other cosmetic additives and non-culinary ingredients were significantly more prevalent in PBMP as compared to MBP (*P* < 0·0001). Concerning nutrient composition, median energy, total fat, saturated fat and protein content were significantly lower, whereas the amounts of carbohydrate, sugar, fibre and salt were significantly higher in PBMP (*P* < 0·05).

**Conclusions:**

Ultra-processing markers are significantly more prevalent in PBMP as compared to MBP. Since UPF intake has been convincingly linked to metabolic and CVD, substituting MBP with PBMP might have negative net health effects.

During recent decades, there has been a considerable shift towards more plant-based dietary patterns^(1)^. Thus, the proportion of 12- to 17-year-old adolescents in Germany following a vegetarian diet more than tripled within 10 years, that is it increased from 1·6 % in 2006 to 5·0 % in 2015 to 2017^(2)^. A plant-based diet not only has the potential to improve human health but also to reduce the impact on the environment as compared to animal-based food products^(3)^. Furthermore, ethical considerations play a major role when choosing a plant-based diet^(1,2)^.

The growing interest in vegetarian diets is leading to an increasing demand for plant-based meat products (PBMP)^(4)^. PBMP replace meat in the human diet and are intended to mimic the texture, taste and appearance of meat^(5,6)^. About two-thirds of the US American population have eaten PBMP in the past year at least once according to a recent survey^(7)^. Interestingly, 22 % and 20 % consumed PBMP daily and at least weekly, respectively^(7)^. The market for PBMP has been growing rapidly worldwide and extends beyond the vegetarian market to include meat-loving consumers who want to reduce their meat consumption for health, environmental and ethical reasons^(8)^. Thus, in the USA the market value of plant-based food products grew from 680 767 to 939 459 $ between 2017 and 2019 corresponding to an 38 % increase in sales over 2 years^(9)^. Future sales of plant-based alternatives are expected to increase globally from $29·4 billion in 2020 to $162 billion by 2030^(10)^.

Previous studies on PBMP focused on potential health benefits by assessing nutrient composition as compared to meat-based products (MBP). Most studies show convincingly that PBMP have a lower energy density^(4,11,12)^, as well as contain less total fat and SFA^(11–15)^ as compared to MBP. In contrast, carbohydrates^(4,12,14–16)^ and dietary fibre^(4,11,12,14–16)^ are higher in PBMP than in MBP. The salt content of PBMP is higher compared to MBP in some^(11,13)^ but not all^(14)^ studies. The amount of protein is the same^(15,16)^ or lower^(11,12,14)^ but protein quality also needs to be considered.

Besides nutrient composition, the extent of processing is an important parameter to evaluate the quality of food items^(17)^. The NOVA classification assesses the extent and purpose of food processing and classifies food products into four groups according to their distance from nature^(17,18)^. Processing according to the NOVA classification includes physical, biological and chemical methods during the manufacturing process, as well as the use of additives^(17,18)^. Cosmetic additives, including flavours, colouring agents and sweeteners, make food products more palatable or appealing^(18)^. Non-culinary ingredients such as fructose, modified oils and protein sources are food substances never or rarely used in the kitchen^(18)^. If an ingredient list contains at least one cosmetic additive or non-culinary ingredient, the product is defined as NOVA group 4, that is ultra-processed food (UPF)^(18)^. Besides the NOVA classification, other systems based on food processing have also been proposed, for example systems suggested by the International Food Information Council Foundation, the International Agency for Research on Cancer, and the National Institute of Public Health in Mexico^(19)^. Compared to these systems, the NOVA classification rates highest in terms of quality since it is most specific, coherent, clear, comprehensive and workable^(19)^.

UPF is ready-to-consume or heat up, and it is usually packaged attractively and marketed intensively^(17)^. It is high in fat, salt and sugar, as well as low in dietary fibre, protein and micronutrients^(17)^. By ultra-processing, products are created that are convenient, hyper-palatable, highly profitable and can replace other food groups^(17)^. Increased consumption of UPF items has been convincingly linked with increased all-cause mortality^(20,21)^, cardiovascular mortality^(21)^, cardiovascular morbidity^(22)^, dementia^(23)^, inflammatory bowel disease^(24)^ and obesity^(25)^. Based on this convincing evidence, avoiding highly processed or UPF has been recommended in several nutrition guidelines including Brazil^(26)^, Canada^(27)^, Chile^(28)^, Japan^(29)^, New Zealand^(30)^, Peru^(31)^ and Uruguay^(32)^.

In the present study, the proportion of ultra-processing is compared between PBMP and MBP overall, as well as in 18 product categories. Furthermore, six ultra-processing bullet categories, that is flavour, flavour enhancer, sweetener, colour, other cosmetic additives and non-culinary ingredients, as well as 33 ultra-processing markers, are assessed in PBMP and MBP using an ingredient list-based approach.

## Methods

All research of PBMP and MBP was performed in the period from March 3, 2022 to May 3, 2022. The study was not registered, and the a priori protocol was not published before conducting the study.

### Plant-based meat product survey and categorisation

A first screen of PBMP was performed onsite in local stores of the top four German food store chains, that is Edeka, Rewe, Lidl and Aldi. Ingredient lists and nutrient composition of these PBMP were extracted. In a second step, the websites of all companies selling PBMP at Edeka, Rewe, Lidl and Aldi were researched online to identify further PBMP not sold in these local food store chains. Ingredient lists and nutrient composition were also extracted from these additional PBMP. PBMP were defined as products actively marketed as MBP replacements, for example vegan/vegetarian minced meat, steak or sausage. Products traditionally used in vegetarian diets and not sold as MBP replacements such as tofu, tempeh and legumes were excluded from the search. However, if these traditional products were part of an actively marketed MBP replacement, for example tofu meat cut or tofu minced meat, they were included in the analysis. A total of 282 PBMP were included.

PBMP were grouped according to their product description, for example meatball, burger or steak. The guiding principles of the German Food Book for meat and meat products^(33)^, as well as for fish, crustaceans and molluscs^(34)^, were used to further specify the categorisation and to group similar meat alternatives into a single category. PBMP that were not listed in the guidelines^(33,34)^, such as the south-eastern European specialty cevapcici, were assigned their own category due to their traditional recipe. If a minimum number of five PBMP were not reached within a category, they were assigned to the product categories ‘Others fish-based’ and ‘Others meat-based’. Using this approach, 18 separate product categories were obtained as shown in Table 1.

**Table 1:** Percentage of ultra-processing and six ultra-processing bullet categories in the total sample, as well as in the 18 product categories, of PBMP and MBP*

Product category	Group	*n*			Ultra-processing bullet categories									
			Ultra-processing		Flavour		Flavour enhancer		Colour		Other cosmetic additives		Non-culinary ingredients	
			%	*n*	%	*n*	%	*n*	%	*n*	%	*n*	%	*n*
Total	MBP	149	52	77	8	12	5	8	1	1	7	11	48	71
	PBMP	282	**88**	**248** §	**70**	**198** §	**21**	**58** §	**17**	**48** §	**63**	**179** §	**77**	**216** §
Minced meat	MBP	10	0	0	0	0	0	0	0	0	0	0	0	0
	PBMP	26	**77**	**20** ‡	**50**	**13** †	19	5	4	1	38	10	**62**	**16** †
Meatball	MBP	10	90	9	20	2	30	3	0	0	40	4	80	8
	PBMP	21	90	19	**81**	**17** †	29	6	5	1	62	13	90	19
Burger	MBP	10	10	1	10	1	10	1	0	0	10	1	10	1
	PBMP	33	**82**	**27** ‡	**61**	**20** †	24	8	3	1	**64**	**21** †	**76**	**25** ‡
Steak	MBP	5	0	0	0	0	0	0	0	0	0	0	0	0
	PBMP	6	67	4	67	4	0	0	17	1	67	4	50	3
Fillet strips	MBP	10	40	4	0	0	0	0	0	0	0	0	40	4
	PBMP	13	**100**	**13** †	**77**	**10** †	38	5	8	1	15	2	85	11
Sausage	MBP	10	70	7	10	1	0	0	0	0	0	0	70	7
	PBMP	26	96	25	**77**	**20** †	12	3	12	3	**69**	**18** ‡	85	22
Kebab	MBP	5	100	5	20	1	20	1	0	0	20	1	100	5
	PBMP	7	86	6	57	4	14	1	0	0	14	1	71	5
Cevapcici	MBP	5	80	4	20	1	0	0	0	0	0	0	80	4
	PBMP	7	86	6	71	5	0	0	0	0	57	4	86	6
Schnitzel	MBP	10	80	8	0	0	0	0	0	0	0	0	80	8
	PBMP	31	97	30	**77**	**24** §	23	7	10	3	**84**	**26** §	90	28
Meat cut	MBP	10	30	3	20	2	20	2	10	1	0	0	10	1
	PBMP	14	64	9	36	5	0	0	0	0	29	4	64	9
Nuggets	MBP	10	70	7	20	2	0	0	0	0	0	0	70	7
	PBMP	25	**100**	**25** †	**80**	**20** †	32	8	8	2	**88**	**22** §	88	22
Salami	MBP	5	40	2	0	0	0	0	0	0	0	0	40	2
	PBMP	9	89	8	**78**	**7** †	44	4	**89**	**8** †	**89**	**8** †	89	8
Lunchmeat	MBP	10	70	7	10	1	10	1	0	0	0	0	70	7
	PBMP	22	73	16	**59**	**13** †	5	1	**41**	**9** †	**68**	**15** †	59	13
Meat paste	MBP	10	70	7	0	0	0	0	0	0	50	5	40	4
	PBMP	10	100	10	**100**	**10** §	40	4	**70**	**7** †	70	7	70	7
Pork sausage	MBP	10	90	9	10	1	0	0	0	0	0	0	90	9
	PBMP	12	83	10	**75**	**9** †	17	2	**50**	**6** †	**75**	**9** †	50	6
Fish fingers	MBP	10	20	2	0	0	0	0	0	0	0	0	20	2
	PBMP	11	**100**	**11** ‡	**91**	**10** ‡	18	2	18	2	**82**	**9** ‡	**73**	**8** †
Others fish-based	MBP	5	0	0	0	0	0	0	0	0	0	0	0	0
	PBMP	5	**100**	**5** †	**100**	**5** †	20	1	40	2	80	4	**100**	**5** †
Others meat-based	MBP	4	50	2	0	0	0	0	0	0	0	0	50	2
	PBMP	4	100	4	50	2	25	1	25	1	50	2	75	3

* Ultra-processing and six ultra-processing bullet categories are presented as percentage and number.

† *P* < 0·05.

‡ *P* < 0·001, and

§ *P* < 0·0001 as assessed by chi-square test.

Values with statistically significant differences as compared to MBP are further indicated in bold.

PBMP, plant-based meat products; MBP, meat-based products.

### Meat-based product survey and categorisation

For all 18 PBMP categories, comparable MBP were researched using the Rewe online store (www.rewe.de) and sorted by popularity. The names of the product categories served as search terms. The number of comparison MBP was based on the number of PBMP as follows: If PBMP within a category were ≥ 10, ten comparison MBP were used; if PBMP within a category were < 10, five comparison MBP were chosen. If the required number of MBP was not reached by search in the Rewe online store, additional sources, that is local Aldi and Lidl stores, as well as the online Bofrost store (www.bofrost.de), were used. Based on this approach, 149 comparison MBP were included.

### Assessment of ultra-processing and nutrient composition

According to Monteiro and co-workers^(17,18)^, 33 ultra-processing markers were identified in English and their German equivalents were researched and adapted as summarised in Supplemental Table 1. Based on these ultra-processing markers, the following six ultra-processing bullet categories were defined: Flavour, flavour enhancer, sweetener, colour, other cosmetic additives and non-culinary ingredients (see online Supplemental Table 1). The ingredient lists for all PBMP and MBP were extracted and analysed concerning ultra-processing markers and bullet categories. If PBMP and MBP were positive for at least one ultra-processing marker, they were regarded as ultra-processed.

All nutritional information to be listed according to the European Union Food Information Regulation No. 1169/2011^(35)^, that is energy in kJ/100 g, as well as fat, saturated fat, carb, sugar, protein and salt in g/100 g, were recorded for all PBMP and MBP. Furthermore, dietary fibre in g/100 g was also captured.

### Additional robustness analyses

Since raw meat product categories are typically non-ultra-processed, they were removed in one set of robustness analyses. More specifically, PBMP and MBP were compared after excluding the product categories of minced meat, burger, steak, fillet strips and meat cut.

An additional onsite robustness analysis was performed at two of the studied food retailers, that is at Rewe (4 Fernie Street, 35 394 Giessen, Germany; *n* PBMP = 87, *n* MBP = 243) and at Lidl (1–3 Georg Elser Street, 35 394 Giessen, Germany; *n* PBMP = 20, *n* MBP = 36). Here, information on all PBMP and all matching MBP was collected, that is the number of matching MBP was not restricted.

### Statistical evaluation

Data were imported, processed, analysed and graphically displayed with R version 4.0.5^(36)^. PBMP and MBP overall and within product categories were compared using chi-square test for categorical variables and Mann-Whitney *U* test for continuous parameters. A *P*-value of < 0·05 was considered as statistically significant in all analyses.

## Results

### Proportion of ultra-processed food items in plant-based meat products and meat-based products

Overall, 282 PBMP were compared to 149 MBP and the main results are summarised in Table 1. The proportion of UPF items was significantly higher in PBMP (88 %) as compared to MBP (52 %) (*P* < 0·0001; Table 1). Within the product categories, the proportion of UPF items was also significantly higher in PBMP *v*. MBP for minced meat (77 % *v*. 0 %), burger (82 % *v*. 10 %), fillet strips (100 % *v*. 40 %), nuggets (100 % *v*. 70 %), fish fingers (100 % *v*. 20 %) and others fish-based (100 % *v*. 0 %) (all *P* < 0·05; Table 1). The proportion of UPF items was numerically but not significantly higher in PBMP *v*. MBP for steak (67 % *v*. 0 %), sausage (96 % *v*. 70 %), cevapcici (86 % *v*. 80 %), schnitzel (97 % *v*. 80 %), meat cut (64 % *v*. 30 %), salami (89 % *v*. 40 %), lunchmeat (73 % *v*. 70 %), meat paste (100 % *v*. 70 %) and others meat-based (100 % *v*. 50 %) (all *P* > 0·05; Table 1). The proportion of UPF items was the same or numerically lower in PBMP *v*. MBP for meatball (90 % *v*. 90 %), kebab (86 % *v*. 100 %) and pork sausages (83 % *v*. 90 %) (all *P* > 0·05; Table 1).

### Ultra-processing bullet categories and markers in plant-based meat products and meat-based products

**Fig. 1:**
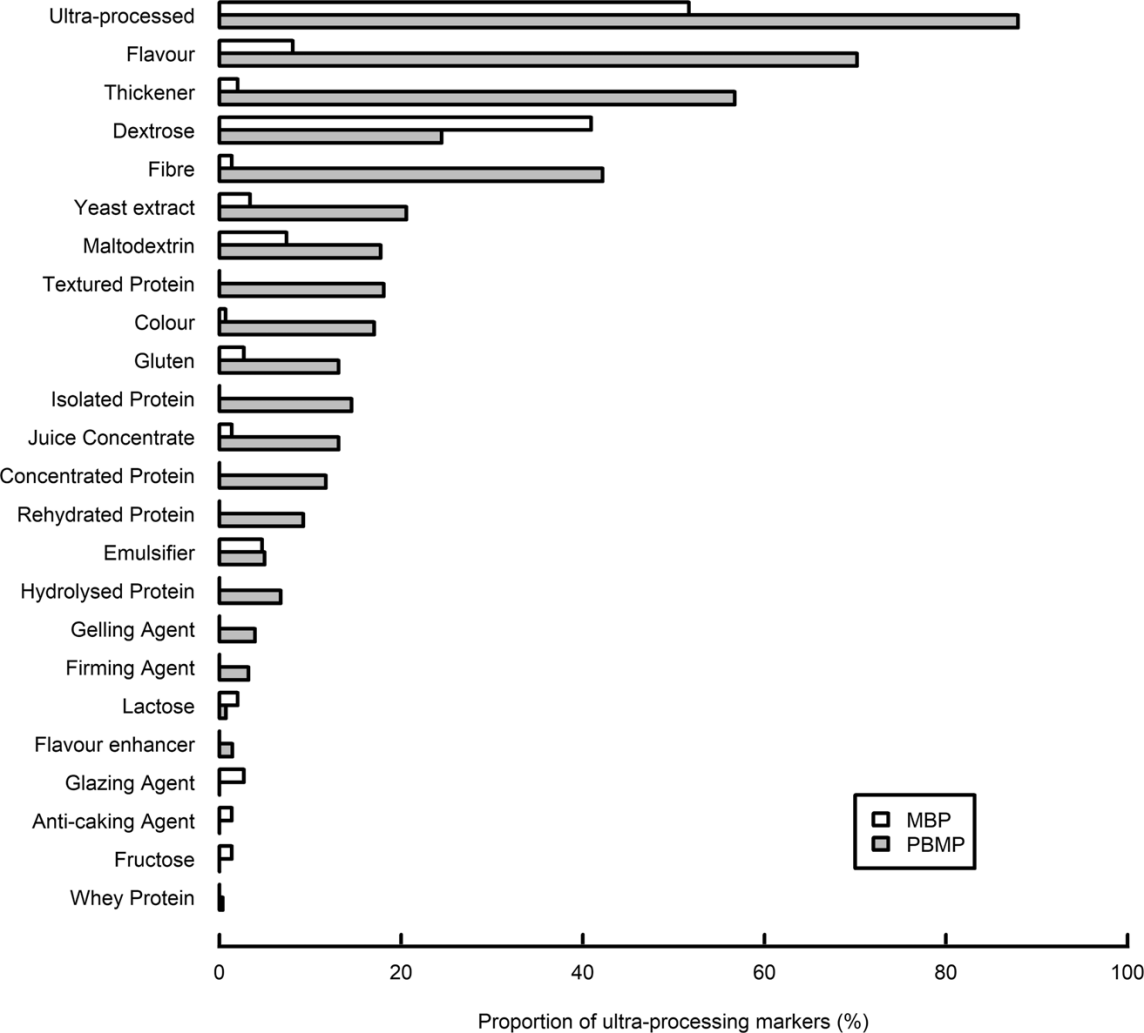
Proportion of ultra-processing markers in MBP (*n* 149) and PBMP (*n* 282). All ultra-processing markers defined in Supplemental Table 1 which were used at least once are depicted

Sweeteners were not found in any PBMP and MBP. Of the remaining five ultra-processing bullet categories, non-culinary ingredients (77 %), flavour (70 %) and other cosmetic additives (63 %) were more frequently detected in PBMP as compared to flavour enhancer (21 %) and colour (17 %) (Table 1). In MBP, non-culinary ingredients was by far the most common ultra-processing bullet category (48 %) followed by flavour (8 %), other cosmetic additives (7 %), flavour enhancer (5 %) and colour (1 %) (Table 1). The proportion of all five ultra-processing bullet categories was significantly higher in PBMP as compared to MBP (*P* < 0·0001, Table 1). In total, 23 out of the 33 ultra-processing markers summarised in Supplemental Table 1 were detected in at least one PBMP or MBP (Fig. 1). Of those, flavour (70 %) and dextrose (41 %) were the most frequently found in PBMP and MBP, respectively (Fig. 1). Of the 23 ultra-processing markers, 18 were more frequently found in PBMP as compared to MBP (Fig. 1).

The proportion of food items with flavour was also significantly higher in PBMP *v*. MBP in 13 out of the 18 product categories, that is minced meat, meatball, burger, fillet strips, sausage, schnitzel, nuggets, salami, lunchmeat, meat paste, pork sausage, fish fingers and others fish-based (all *P* < 0·05; Table 1). PBMP did not show a significantly higher percentage of flavour enhancer compared to MBP in any of the 18 product categories. The share of colour in PBMP *v*. MBP was significantly higher in salami, lunchmeat, meat paste, and pork sausage (all *P* < 0·05; Table 1). For other cosmetic additives, eight product categories showed a significantly higher proportion in PBMP as compared to MBP, that is burger, sausage, schnitzel, nuggets, salami, lunchmeat, pork sausage and fish fingers (all *P* < 0·05; Table 1). The proportion of items with non-culinary ingredients was significantly higher in PBMP *v*. MBP in four product categories, that is minced meat, burger, fish fingers and others fish-based (all *P* < 0·05; Table 1).

### Nutrient composition of plant-based meat products and meat-based products

Median (range) values for the nutrient composition of PBMP and MBP are summarised in Table 2. Median energy (880·5 *v*. 972·0 kJ/100 g), total fat (11·0 *v*. 15·8 g/100 g), saturated fat (1·2 *v*. 4·0 g/100 g) and protein (14·1 *v*. 17·0 g/100 g) contents of the PBMP were significantly lower than the values of the MBP (all *P* < 0·05; Table 2). In contrast, the amounts of carbohydrate (7·1 *v*. 1·0 g/100 g), sugar (1·5 *v*. 0·5 g/100 g), fibre (4·5 *v*. 0·3 g/100 g) and salt (1·6 *v*. 1·3 g/100 g) were significantly higher in PBMP as compared to MBP (all *P* < 0·05; Table 2). There was significant heterogeneity in nutrient composition between PBMP and MBP within the 18 different product categories (Table 2).

**Table 2:** Nutrient composition in the total sample and in the 18 product categories, of PBMP and MBP*

Product category	Group	*n*	Energy		Fat		Saturated fat		Carb		Sugar		Fibre		Protein		Salt	
			Median	Range	Median	Range	Median	Range	Median	Range	Median	Range	Median	Range	Median	Range	Median	Range
Total	MBP	149	972·0	410·0–2107·0	15·8	1·0–45·0	4·0	0·0–19·0	1·0	0·0–28·4	0·5	0·0–8·9	0·3	0·0–5·6	17·0	6·9–25·0	1·3	0·0–4·2
	PBMP	282	**880·5**	**132·0–1592·0** †	**11·0**	**0**·**5–33·0** §	**1·2**	**0·1–17·7** §	**7·1**	**0·8–27·6** §	**1·5**	**0·0–10·0** §	**4·5**	**0·7–20·1** §	**14·1**	**1·3–62·0** §	**1·6**	**0·0–6·2** †
Minced meat	MBP	10	972·0	734·0–1018·0	18·0	11·0–19·0	7·0	0·0–8·1	0·0	0·0–0·5	0·0	0·0–0·3	0·0	0·0–0·5	18·0	18·0–20·0	0·0	0·0–0·2
	PBMP	26	840·5	441·0–1407·0	**8·5**	**0·5–20·6** ‡	**1·1**	**0·1–16·0** †	**5·8**	**0·9–27·0** §	**1·6**	**0·0–8·7** §	**5·3**	**1·3–20·1** §	18·2	6·4–53·8	**1·4**	**0·0–6·2** §
Meatball	MBP	10	1085·0	410·0–1226·0	20·5	7·1–22·0	8·0	3·1–9·8	7·3	0·7–10·0	0·9	0·3–2·1	2·1	2·1–2·1	14·5	6·9–17·0	1·8	1·2–2·3
	PBMP	21	881·0	132·0–1300·0	**11·9**	**1·7–18·0** †	**1·5**	**0·2–7·4** §	8·3	1·0–21·4	**2·0**	**0·1–6·4** †	4·0	1·0–8·2	16·0	2·6–22·0	**1·4**	**0·3–2·0** †
Burger	MBP	10	1042·0	582·0–1246·0	20·0	1·7–21·9	8·2	0·2–9·5	0·1	0·0–28·4	0·0	0·0–2·9	0·0	0·0–0·0	17·1	9·7–18·0	1·1	0·9–1·7
	PBMP	33	**826·0**	**511·0–1134·0** †	**10·0**	**2·6–19·0** ‡	**1·3**	**0·3–11·0** †	**6·5**	**1·8–22·0** †	**1·3**	**0·0–6·7** †	**4·6**	**1·3–9·7** †	14·0	5·9–29·0	**1·5**	**0·8–2·1** †
Steak	MBP	5	505·0	440·0–559·0	4·0	2·0–5·0	1·8	0·8–2·1	0·0	0·0–0·0	0·0	0·0–0·0	0·0	0·0–0·0	21·5	21·0–22·0	0·0	0·0–0·2
	PBMP	6	**917·5**	**528·0–1056·0** †	8·2	1·2–11·0	1·4	0·2–4·8	**7·6**	**6·5–27·0** †	**2·4**	**1·2–8·6** †	**4·8**	**4·5–5·4** †	17·7	13·0–49·0	**1·3**	**0·0–1·6** †
Fillet strips	MBP	10	477·0	443·0–607·0	2·0	1·0–4·0	0·6	0·0–2·0	0·1	0·0–5·0	0·1	0·0–2·0	0·0	0·0–0·1	23·0	22·0–23·9	0·2	0·0–3·0
	PBMP	13	**714·0**	**477·0–1497·0** †	**5·3**	**1·2–17·0** †	0·5	0·3–1·9	**3·3**	**1·1–19·0** †	0·5	0·0–4·4	**6·8**	**2·5–9·8** ‡	**18·0**	**9·7–62·0** †	**1·3**	**0·6–2·5** †
Sausage	MBP	10	1152·0	941·0–1443·0	24·0	18·0–32·0	9·0	6·0–13·4	1·0	0·0–1·0	0·7	0·0–1·0	0·1	0·0–1·0	14·1	13·0–18·0	2·0	1·7–2·3
	PBMP	26	**831·0**	**481·0–1356·0** †	**13·1**	**6·6–24·4** §	**2·9**	**0·8–17·7** ‡	**4·8**	**2·3–12·0** §	0·8	0·0–3·0	**4·8**	**0·7–7·2** ‡	14·3	2·0–27·2	**1·7**	**1·0–5·3** †
Kebab	MBP	5	828·0	615·0–1345·0	11·4	7·8–24·0	3·2	1·0–5·6	3·4	1·4–5·7	1·0	0·7–2·4	0·4	0·4–0·4	18·5	14·0–25·0	1·7	1·3–2·7
	PBMP	7	799·0	544·0–1065·0	**6·9**	**3·9–14·0** †	**0·8**	**0·5–1·3** †	3·9	0·8–9·0	0·7	0·0–4·6	4·7	0·8–6·2	27·0	15·0–27·8	1·9	1·2–2·5
Cevapcici	MBP	5	1036·0	938·0–1078·0	19·5	14·0–20·0	9·3	3·3–10·6	1·0	1·0–5·6	0·5	0·5–1·1	0·9	0·9–0·9	16·0	14·5–23·0	1·5	1·3–2·5
	PBMP	7	972·0	654·0–1277·0	**16·0**	**9·0–18·0** †	5·0	1·8–8·6	**6·5**	**2·5–15·0** †	1·4	0·1–4·4	5·7	4·3–6·0	14·0	1·5–29·0	2·0	1·6–2·3
Schnitzel	MBP	10	933·5	691·0–979·0	10·4	3·6–12·0	2·2	0·9–3·9	16·2	11·0–19·5	1·2	0·5–6·7	0·6	0·4–0·9	16·7	12·9–17·2	1·3	0·9–1·4
	PBMP	31	938·0	353·0–1272·0	11·0	1·6–19·1	**1·2**	**0·4–3·8** †	17·0	1·9–26·1	1·3	0·0–5·1	**4·1**	**2·0–8·3** †	**13·1**	**5·3–20·0** †	**1·4**	**0·8–2·0** †
Meat cut	MBP	10	578·9	470·0–650·0	4·5	1·4–9·3	1·7	0·0–3·7	0·6	0·0–9·0	0·1	0·0–8·9	0·0	0·0–0·4	21·4	16·5–24·4	0·8	0·0–1·1
	PBMP	14	**832·5**	**417·0–1407·0** †	6·5	1·2–19·0	1·0	0·3–3·3	**3·3**	**1·2–27·0** ‡	**1·1**	**0·0–8·6** †	**4·5**	**1·0–7·1** ‡	26·0	9·7–49·0	**1·6**	**0·0–3·4** ‡
Nuggets	MBP	10	1031·0	867·0–1239·0	13·5	11·0–20·0	3·0	1·5–7·9	18·4	2·7–22·0	0·7	0·4–2·1	0·8	0·0–1·1	15·0	9·7–22·0	1·0	0·8–2·1
	PBMP	25	**918·0**	**487·0–1144·0** †	**9·9**	**3·1–16·0** †	**0·9**	**0·3–2·3** §	16·1	4·4–27·6	1·6	0·0–10·0	**4·0**	**2·8–12·0** †	13·0	1·3–23·0	**1·5**	**1·0–2·2** †
Salami	MBP	5	1461·0	1269·0–1552·0	28·0	24·0–30·0	11·0	8·6–12·0	1·0	1·0–1·0	1·0	1·0–1·0	0·5	0·0–1·0	24·0	19·0–24·5	4·0	3·5–4·1
	PBMP	9	**759·0**	**559·0–1075·0** †	**11·0**	**9·1–19·0** †	**0·8**	**0·7–5·2** †	**6·0**	**4·8–8·5** †	1·5	0·8–4·8	3·2	2·8–3·2	11·5	4·0–29·0	**2·6**	**1·2–2·9** †
Lunchmeat	MBP	10	704·0	416·0–1162·0	10·8	1·2–26·0	3·2	0·4–17·0	1·0	0·5–1·2	0·8	0·3–1·2	0·5	0·3–0·5	16·7	11·0–21·0	2·1	2·0–2·8
	PBMP	22	739·5	525·0–1078·0	11·9	5·2–16·0	0·9	0·6–4·8	**4·9**	**3·0–9·3** §	**3·1**	**1·4–3·9** §	**2·5**	**0·8–7·7** ‡	8·4	2·2–29·6	2·0	1·0–3·0
Meat paste	MBP	10	1396·5	1084·0–1771·0	31·0	21·0–42·0	12·7	6·3–16·0	0·7	0·5–3·5	0·6	0·3–1·4	0·5	0·3–5·6	14·0	11·0–15·5	2·0	1·4–2·8
	PBMP	10	**998·0**	**728·0–1094·0** ‡	**22·4**	**12·0–25·5** ‡	**1·6**	**1·3–3·6** ‡	**5·2**	**2·9–15·0** ‡	0·9	0·3–3·7	3·7	1·9–6·0	**2·7**	**2·5–6·2** ‡	1·9	1·5–2·5
Pork sausage	MBP	10	1183·5	957·0–2107·0	26·0	20·0–45·0	10·5	6·0–19·0	1·0	0·5–1·1	0·7	0·5–1·1	0·5	0·0–0·5	12·0	11·0–25·0	2·1	1·8–4·2
	PBMP	12	**844·5**	**525·0–1592·0** †	**14·9**	**5·2–33·0** †	**1·1**	**0·6–2·6** §	**5·7**	**1·1–8·0** §	**2·8**	**1·0–3·9** ‡	**2·4**	**1·0–5·5** †	8·4	4·0–29·6	2·3	1·4–3·5
Fish fingers	MBP	10	824·0	759·0–1034·0	8·2	7·7–14·0	1·0	0·6–1·2	17·8	13·4–20·5	0·9	0·5–2·0	0·9	0·8–1·1	13·0	11·0–13·5	0·9	0·7–1·2
	PBMP	11	**1029·0**	**822·0–1289·0** †	**11·9**	**7·3–20·0** †	**1·1**	**0·9–2·6** †	21·0	2·6–26	1·0	0·4–3·1	**3·2**	**2·0–8·4** †	11·0	2·9–15·7	**1·3**	**0·7–1·7** †
Others fish-based	MBP	5	815·0	439·0–924·0	11·0	1·3–15·7	2·0	0·2–3·3	0·0	0·0–0·3	0·0	0·0–0·3	0·0	0·0–0·0	21·0	20·0–24·0	0·5	0·1–3·1
	PBMP	5	864·0	543·0–1160·0	13·0	7·1–19·9	1·7	0·9–2·8	**6·8**	**1·7–8·6** †	**0·5**	**0·1–1·3** †	2·9	1·1–4·6	11·0	2·7–22·6	1·1	0·9–1·8
Others meat-based	MBP	4	989·0	448·0–1304·0	17·8	2·0–25·8	6·4	1·0–8·2	0·8	0·0–1·0	0·5	0·0–0·9	0·3	0·0–0·5	19·8	17·0–22·0	0·5	0·0–4·0
	PBMP	4	980·0	324·0–1282·0	15·0	0·5–20·0	2·6	0·1–9·0	**8·5**	**1·3–9·6** †	2·5	0·5–6·8	3·6	3·2–4·0	17·0	12·0–22·0	1·9	1·3–2·9

* Variables for the nutrient composition are presented as median and range. Fibre content was not given for all food items, and it was indicated for only one item within Meatball-, Kebab- and Cevapcici-MBP.

† *P* < 0·05.

‡ *P* < 0·001 and

§ *P* < 0·0001 as assessed by Mann-Whitney *U* test.

Values with statistically significant differences as compared to MBP are further indicated in bold.

PBMP, plant-based meat products; MBP, meat-based products.

### Additional robustness analyses

If raw meat product categories were removed from the analysis, the proportions of UPF items, flavour, flavour enhancer, colour, other cosmetic additives and non-culinary ingredients remained all significantly higher in PBMP as compared to MBP (all *P* < 0·05; see online Supplemental Table 2).

In the onsite robustness analyses, the proportions of UPF items, flavour and other cosmetic additives were significantly higher in PBMP as compared to MBP at both Rewe and Lidl (all *P* < 0·05; see online Supplemental Table 3). Flavour enhancer, colour and non-culinary ingredients were all more prevalent in PBMP as compared to MBP with differences reaching statistical significance at Rewe (all *P* < 0·0001) but not at Lidl (*P* > 0·05) (see online Supplemental Table 3). Energy, total fat, saturated fat and protein contents were significantly lower, and carbohydrate and fibre amounts were significantly higher in PBMP as compared to MBP at both supermarkets onsite (all *P* < 0·05; see online Supplemental Table 4).

## Discussion

### Principal findings

The present study systematically assesses the extent of ultra-processing, as well as ultra-processing bullet categories and ultra-processing markers, in PBMP and their meat-based counterparts. We demonstrate that about nine out of ten PBMP fulfil ultra-processing criteria according to the NOVA classification in contrast to about half of the MBP. Of the 18 product categories examined, 15 show numerically higher proportions of ultra-processing for PBMP as compared to MBP. All ultra-processing bullet categories which are present in the studied products, that is flavour, flavour enhancer, colour, other cosmetic additives and non-culinary ingredients, are more frequently observed in PBMP than in MBP. Of 23 ultra-processing markers present in the products, 18 are detected in higher proportions in PBMP as compared to MBP. Concerning nutrient composition, median energy, total fat, saturated fat and protein content are significantly lower, whereas the amounts of carbohydrate, sugar, fibre and salt are significantly higher in PBMP as compared to MBP. Combined these findings suggest that a much higher proportion of PBMP fulfil ultra-processing criteria as compared to their meat-based counterparts whereas some aspects of the nutrient composition of PBMP appear favourable including higher fibre amounts, as well as lower energy, fat and SFA content.

### Comparison with other studies

In an analysis comprising 148 PBMP sold by seven of the most common supermarket chains in Spain, the proportion of PBMP in NOVA group 4 is 94 %^(37)^ which is similar to the 88 % found in the current analysis. In another study from Spain combining 198 PBMP and 33 plant-based dairy products within one analysis and using data from Open Food Facts, a lower proportion, that is 59 % of the plant-based foods with a NOVA classification label, is NOVA group 4^(38)^. However, for 63 % of the plant-based foods in this study, no information concerning NOVA classification is available^(38)^.

Various reports have elucidated the intake of UPF in vegetarians and vegans as compared to meat eaters. In a study conducted on 21 212 participants from the prospective observational NutriNet-Santé cohort in France between 2014 and 2018, higher avoidance of animal-based foods is associated with a higher consumption of UPF^(39)^. Thus, the proportions of energy intake from UPF in relation to total energy intakes are 33·0 %, 32·5 %, 37·0 % and 39·5 % for meat eaters, pesco-vegetarians, vegetarians and vegans, respectively^(39)^. However, standard deviations are rather large and no post hoc tests are presented besides the ANOVA result (*P* < 0·0001) to elucidate which group means differ from one another significantly^(39)^. In agreement with these findings, both healthy and unhealthy eating patterns exist in a convenience sample of 129 vegans^(40)^. Two clusters, that is ‘convenience’ and ‘traditional’ are identified that consist of an array of ultra-processed vegan food items and represent almost half of the participants^(40)^. In a German sample of 814 participants, PBMP consumption is predominant within a vegetarian diet while other ultra-processed product groups such as convenience, fast foods, snacks and ultra-processed beverages are mainly consumed by meat eaters^(41)^. Of note, consumption of all types of UPF is lowest in flexitarians^(41)^. Taking these published and the current data into consideration, different dietary patterns exist in vegetarians and vegans. A recent systematic review demonstrates convincingly that vegetarian and vegan diets have a higher overall diet quality^(42)^. However, there are some dietary patterns in vegetarians and vegans that show higher UPF consumption than omnivores, and PBMP might contribute to this increased UPF intake.

A higher UPF intake has been convincingly linked to adverse outcomes^(20–25)^. Moreover, flavours as the most prevalent ultra-processing marker in PBMP might induce overeating and body weight gain, thereby, contributing to the obesity epidemic^(43)^. Taking these studies into consideration, it is well possible that PBMP consumption might have adverse effects on metabolic and cardiovascular endpoints due to a higher proportion of ultra-processing. However, some aspects of PBMP nutrient composition appear favourable in the current analysis including higher fibre amounts and lower energy content as compared to MBP which is in accordance with the majority of published evidence^(44,45)^. Salt content is increased in the current analysis of PBMP which has also been described in various reports^(44)^. It needs to be elucidated in future analyses how increased ultra-processing and altered nutrient composition affect the nutritional quality of PBMP as compared to MBP. The current study supports recent evidence that plant-based diets are not necessarily healthy^(39–41,46)^. Besides ultra-processing and nutrient composition, further aspects of PBMP need to be assessed in future studies which include improving current production techniques, climate change and changing demographics^(47,48)^.

### Strength and limitations of this study

The present study systematically assesses ultra-processing bullet categories and ultra-processing markers in a broad range and variety of PBMP and MBP. Further strengths include that all PBMP are compared to their respective meat-based counterparts from the same local stores and that a search term-based approach according to the NOVA classification is used.

However, the study has some limitations. Thus, all assessments are performed exclusively for the German market and the composition of PBMP and MBP might differ in other regions. Furthermore, some product categories are rather small which affects the statistical power. Moreover, dietary fibre data in PBMP and MBP are incomplete since labelling is optional according to the German food law^(35)^.

Various factors might introduce bias in favour of the MBP: Although PBMP sampling includes two discounters (Lidl and Aldi), MBP are sampled mostly from one relatively upmarket online supermarket (Rewe) which may offer a healthier product range than the cheaper discounters. Furthermore, the popularity ranking in the Rewe online store depends on the location from which the website is accessed and healthy foods might rank higher in affluent areas with many young, health-conscious consumers. Moreover, the range of foods offered in the online store might systematically be different from the brick-and-mortar ones. In addition, limiting the number of MBP as comparators to PBMP might introduce selection bias. However, independent robustness analyses examining all available PBMP and all matching MBP onsite under identical conditions at Rewe and Lidl show results comparable to the current findings. Furthermore, results remain similar if PBMP are compared to MBP excluding raw meat. Products traditionally used in vegetarian diets such as tofu, tempeh and legumes are frequently used as PBMP but are excluded from the current analysis as long as they are not marketed as MBP replacements which introduces further bias in favour of MBP.

The approach used in the current manuscript to identify UPF most closely resembles the ingredient marker method described by Ricardo and co-workers^(49)^. However, it has been convincingly demonstrated that the detection of UPF items differs depending on the approach used and the selection of individual ultra-processing markers^(49)^.

### Conclusions

The current study indicates that the proportion of UPF items is higher in PBMP as compared to MBP overall, as well as in various product categories. In contrast, some aspects of the macronutrient composition of PBMP appear favourable including higher fibre amounts, as well as lower energy, fat and SFA content. Since UPF intake has been convincingly linked to metabolic and CVD, substituting MBP with PBMP might have negative net health effects.

## Supporting information

Metz et al. supplementary material Metz et al. supplementary material

## Supplementary material

For supplementary material accompanying this paper visit https://doi.org/10.1017/S1368980023002458
