# Supplementary material for: Ultra-processing markers are more prevalent in plant-based meat products as compared to their meat-based counterparts in a German food market analysis
Source: Public Health Nutr. 2023 Nov 6;26(12):2728–37. doi: 10.1017/S1368980023002458 (PMC10755440; doi:10.1017/S1368980023002458)
Supplement: Metz et al. supplementary material [file S1368980023002458sup001.docx]

**Supplementary Table 1**

Search terms for the six ultra-processing bullet categories and 33 ultra-processing markers^1^

| **Ultra-processing** **bullet categories** | **Ultra-processing markers in**  **English** | | **Ultra-processing markers in German** |
| --- | --- | --- | --- |
| Flavour | 1 | Flavour | (A\|a)rom(a\|en) |
| Flavour enhancer | 2  3 | Flavour enhancer  Yeast (extract\|flake)^#^ | Geschmacksverstärker  Hefe(extrakt\|flocken) |
| Sweetener | 4 | Sweetener | Sü(ß\|ss)ungsmittel |
| Colour | 5 | Colour | Farbstoff |
| Other cosmetic additives | 6  7  8  9  10  11  12  13  14  15  16 | Carbonating agent  Firming agent  Bulking agent  Defoaming agent  Anti-caking agent  Glazing agent  Emulsifier  Sequestrant  Humectant  Thickener  Gelling agent | Kohlendioxid  Festigungsmittel  Füllstoff  Schaummittel  Trennmittel  Überzugsmittel  Emulgator  Komplexbildner  Feuchthaltemittel  Verdickungsmittel  Geliermittel |
| Non-culinary ingredients | 17  18  19  20  21  22  23  24  25  26  27  28  29  30  31  32  33 | Fructose  Fruit juice concentrate  Invert sugar  Maltodextrin  Dextrose  Lactose  Fibre  Hydrogenated oil  Hydrolysed protein  Rehydrated protein^#^  Textured protein^#^  Protein isolate^#^  Protein concentrate^#^  Gluten  Casein  Whey protein  Mechanically separated  meat | Fru(c\|k)tose, Fruchtzucker  (S\|s)aftkonzentrat  Invertzucker  Maltodextrin  Dextrose  La(c\|k)tose, Milchzucker  (F\|f)aser  Gehärtete Fette  Hydrol(i\|y)siert  Rehydriert  (T\|t)extur(at\|iert)  ((E\|e)iweiß\|(P\|p)rotein)isolat  ((E\|e)iweiß\|(P\|p)rotein)konzentrat  (G\|g)luten,Weizenkleber  (C\|K)asein  Molkenprotein  Separatorenfleisch |

^1^English search terms are based on NOVA group 4^(17,18)^ and the indicated corresponding German search terms were used for the analysis. For various English search terms, more than one German equivalent exists and these equivalents are listed with the logical operator | indicating OR, i.e., the search term (A|a)rom(a|en) identifies words including “Aroma”, “Aromen”, “aroma” (like in “Raucharoma”), and “aromen” (like in “Raucharomen”). ^#^Indicates search terms which are not literally mentioned in the publications by Monteiro and co-workers^(17,18)^ but which are related to the ultra-processing markers mentioned.

**Supplementary Table 2**

Percentage of ultra-processing and six ultra-processing bullet categories in the total sample of PBMP and MBP excluding raw meat, i.e., excluding the product categories minced meat, burger, steak, fillet strips, and meat cut.^1^

| **Product category** | **Group** | **Ultra-processing** | **Ultra-processing bullet categories** | | | | |
| --- | --- | --- | --- | --- | --- | --- | --- |
|  |  |  | **Flavour** | **Flavour**  **enhancer** | **Colour** | **Other cosmetic additives** | **Non-culinary ingredients** |
|  | (n) | %  (n) | %  (n) | %  (n) | %  (n) | %  (n) | %  (n) |
| Total | MBP  (104) | 66  (69) | 9  (9) | 5  (5) | 0  (0) | 10  (10) | 63  (65) |
|  | PBMP  (190) | **92**  **(175)^†^** | **77**  **(146)^†^** | **21**  **(40)^§^** | **23**  **(44)^†^** | **73**  **(138)^†^** | **80**  **(152)^#^** |

^1^Ultra-processing and six ultra-processing bullet categories are presented as percentage (number). ^#^p < 0.05, ^§^p < 0.001, and ^†^p < 0.0001 as assessed by chi-square test. Values with statistically significant differences as compared to MBP are further indicated in bold.

**Supplementary Table 3**

Percentage of ultra-processing and six ultra-processing bullet categories of PBMP and MBP at Rewe and Lidl onsite markets^1^

| **Product category** | **Group** | **Ultra-processing** | **Ultra-processing bullet categories** | | | | |
| --- | --- | --- | --- | --- | --- | --- | --- |
|  |  |  | **Flavour** | **Flavour**  **enhancer** | **Colour** | **Other cosmetic additives** | **Non-culinary ingredients** |
|  | (n) | %  (n) | %  (n) | %  (n) | %  (n) | %  (n) | %  (n) |
| Rewe | MBP  (243) | 62  (150) | 7  (17) | 3  (7) | 3  (8) | 13  (31) | 58  (141) |
|  | PBMP  (87) | **100**  **(87)^†^** | **95**  **(83)^†^** | **20**  **(17)^†^** | **26**  **(23)^†^** | **68**  **(59)^†^** | **86**  **(75)^†^** |
| Lidl | MBP  (36) | 67  (24) | 3  (1) | 0  (0) | 6  (2) | 14  (5) | 67  (24) |
|  | PBMP  (20) | **100**  **(20)^#^** | **90**  **(18)^†^** | 5  (1) | 15  (3) | **90**  **(18)^†^** | 90  (18) |

^1^Ultra-processing and six ultra-processing bullet categories are presented as percentage (number). ^#^p < 0.05, ^§^p < 0.001, and ^†^p < 0.0001 as assessed by chi-square test. Values with statistically significant differences as compared to MBP are further indicated in bold.

**Supplementary Table 4**

Nutrient composition of PBMP and MBP at Rewe and Lidl onsite markets^1^

| **Product category** | **Group**  (n) | **Energy**  median  (range) | **Fat**  median  (range) | **Saturated fat**  median  (range) | **Carb**  median  (range) | **Sugar**  median  (range) | **Fibre**  median  (range) | **Protein**  median  (range) | **Salt**  median  (range) |
| --- | --- | --- | --- | --- | --- | --- | --- | --- | --- |
| Rewe | MBP  (243) | 987.0  (215 to  2167.0) | 19.0  (0.6 to  47.0) | 6.0  (0.0 to  19.0) | 1.0  (0.0 to  24.0) | 0.5  (0.0 to  8.3) | 0.3  (0.0 to  5.6) | 18.0  (4.1 to  34.0) | 2.1  (0.0 to  4.6) |
|  | PBMP  (87) | **840.0**  **(265.0 to**  **1306.0)^†^** | **11.0**  **(0.5 to**  **30.0)^†^** | **1.1**  **(0.1 to**  **8.7)^†^** | **6.0**  **(0.0 to**  **27.0)^†^** | **1.2**  **(0.0 to**  **4.5)^†^** | **5.7**  **(1.3 to**  **9.6)^†^** | **12.0**  **(0.6 to**  **32.0)^†^** | **1.6**  **(0.8 to**  **3.5)^#^** |
| Lidl | MBP  (36) | 993.0  (99.0 to 1463.0) | 19.0  (3.3 to  34.5) | 7.2  (0.5 to  12.2) | 1.2  (0.0 to  19.0) | 1.0  (0.0 to  5.6) | 0.8  (0.0 to  0.9) | 15.0  (4.9 to  22.9) | 1.6  (0.1 to  2.3) |
|  | PBMP  (20) | **816.0**  **(314.0 to 1235.0)^#^** | **10.5**  **(1.5 to 26.1)^§^** | **1.1**  **(0.1 to**  **4.0)^†^** | **7.1**  **(0.0 to**  **30.6)^#^** | 1.4  (0.0 to  6.5) | **4.7**  **(2.9 to**  **7.9)^#^** | **8.6**  **(1.6 to**  **19.0)^§^** | **1.8**  **(0.9 to**  **2.6)^#^** |

^1^Variables for nutrient composition are presented as median (range). Fibre content was not given for all food items. ^#^p < 0.05, ^§^p < 0.001, and ^†^p < 0.0001 as assessed by Mann-Whitney U test. Values with statistically significant differences as compared to MBP are further indicated in bold.
